# A survey on dogs with valvular disease flying to Japan for operation

**DOI:** 10.1038/s41598-023-29476-1

**Published:** 2023-03-27

**Authors:** Arane Takahashi, Sayaka Takeuchi, Ayaka Chen, Masami Uechi

**Affiliations:** JASMINE Veterinary Cardiovascular Medical Center, 1-8-37 Nakagawa Tsuzuki-ku, Yokohama, Kanagawa 224-0001 Japan

**Keywords:** Cardiovascular biology, Cardiovascular diseases, Valvular disease

## Abstract

In small-breed dogs, myxomatous mitral valve disease (MMVD) is a common disease which may lead to chronic heart failure. Mitral valve repair is an optimal surgical treatment that is currently available in limited veterinary facilities globally because it requires a special surgery team and specific devices. Therefore, some dogs must travel overseas to undergo this surgery. However, a question arises regarding the safety of dogs when traveling by air with a heart disease. We aimed to evaluate the effect of flight journey on dogs with mitral valve disease, including survival rates, symptoms during the trip, laboratory test results, and operational outcomes. All dogs stayed near the owner in the cabin during the flight. The survival rate after the flight was 97.5% in 80 dogs. The surgical survival rates (96.0% and 94.3%) and hospitalization periods (7 days and 7 days) were similar between overseas and domestic dogs. This report shows that taking air flights in the cabin may not have a significant effect on dogs with MMVD, on the premise that their overall conditions are stable under cardiac medication.

## Introduction

Myxomatous mitral valve disease (MMVD) is a common progressive cardiac disease in small-breed dogs^1,2^. Mitral valve repair (MVR) is a promising surgical treatment^3^ and requires facilities equipped with extracorporeal circulation and a trained expert team, which are still limited globally. As is the case, some dogs with MMVD must travel overseas to undergo MVR. Flight by an aircraft can trigger unfavorable health situations in humans with cardiac disease^4–6^, but no studies have referred to the risks of air travel for dogs with MMVD. This study aimed to evaluate the risk and effect of an international flight on dogs with MMVD. Among the dogs that underwent MVR, the operational survival rate and hospitalization period were compared to those of domestic dogs during the same period.

## Methods

Dogs diagnosed with MMVD that took oversea air flights in passenger cabins to undergo MVR in Japan between September 2017 and March 2019 were included. Dogs were excluded from the study if they had previously undergone MVR. For the inbound survival rate, deaths of dogs during the period from the first takeoff until the day of the MVR were counted. The dog’s condition and symptoms observed during the inbound trip were obtained using a questionnaire administered to the owners. The owners were not notified beforehand of the questionnaire contents to avoid preconceptions. Questions were asked whether the owner recognized clinical conditions, such as a decrease in activity level or appetite, presence of vomiting, diarrhea, dyspnea, or coughing, compared to the dog’s base status before getting on board. Occurrence rates were calculated for each clinical condition. Dogs were excluded from the calculation if the questionnaire was incomplete, if the owners were not near the dog during the flight, or if the dog was sedated during the flight. Blood tests were conducted to assess dehydration (to determine if the following two of the three values were met: hematocrit > 55.0%, total protein > 7.2 g/dl, or blood urea nitrogen > 29.2 mg/dl), azotemia (blood urea nitrogen > 29.2 mg/dl and creatinine > 1.4 mg/dl), and systemic inflammation (C-reactive protein > 0.7). The cut-off value for each parameter was set based on the manufacturer’s standards, and thoracic radiography was performed to calculate the vertebral heart size (VHS) and assess pulmonary exudates. Echocardiography was performed by a trained specialist for the following measurements: left atrial to aortic ratio (LA/Ao) and left ventricular end-diastolic internal diameter normalized for body weight (LVIDDn). Cardiac enlargement was defined as VHS > 10.5, LA/Ao ≥ 1.6, and LVIDDn ≥ 1.7, based on ACVIM consensus guidelines^6^. This guideline, revised by the ACVIM Specialty of Cardiology Consensus Panel, classifies MMVD and heart failure. Written informed consent was obtained from all dog owners for the use of data for publication. The preoperative clinical laboratory values for each dog were compared to the most recent medical record obtained from local veterinarians prior to air flight. Dogs were excluded if medical records from their homeland could not be obtained or if the most recent record was not within 4 months prior to the preoperative examination.

This study was approved by the Institutional Animal Care and Use Committee of the JASMINE Veterinary Cardiovascular Medical Center which is in compliance with the ARRIVE guidelines (approval code: 2018–9). All the procedures were in accordance with the institutional guideline in terms of animal welfare. All clinical data of the dogs in this study was used from the examinations necessary for their original purpose of the animal hospital visit, i.e. cardiac surgery.

## Results

### Inbound survival rate and observed symptoms

Eighty-two dogs flew overseas during the study period to undergo MVR in Japan from September 2017 to March 2019. All dogs were in the passenger cabin during the flight. No quarantine period was necessary as they had fulfilled the requirements to enter Japan including a 6-month quarantine in their own country prior to the flight. Two dogs were excluded from the study because they underwent a second MVR. The numbers of dogs were as follows: Chihuahua 14, Maltese 15, Cavalier King Charles Spaniel 10, Mixed breed 10, Shih Tzu 9, Miniature Schnauzer 4, Toy Poodle 3, Yorkshire Terrier 3, Papillon 2, Pomeranian 2, American Eskimo 1, Bichon Frise 1, Beagle 1, Chinese Crested Dog 1, Dachshund 1, Havanese 1, Pekingese 1, and Spitz 1. The characteristics of the dogs, grouped by departure region, are summarized in Table 1.

**Table 1:** Profile of dogs grouped by regions of their destination.

	Asia	Americas	Europe	All
Flight hours	1.5–5	7.5–21	11–17	–
Number of dogs	19	57	4	80
Age (years)	9.6 ± 1.5	10.0 ± 1.9	9.7 ± 1.5	9.9 ± 1.8
Body weight (kg)	3.8 ± 1.2	5.5 (2.3–15.0)	7.9 ± 7.3	5.1 (2.1–18.6)
Sex, male/female [%]	16/3 [84.2/15.8]	36/21 [63.2/36.8]	3/1 [75/25]	55/25 [69/31]
ACVIM* B1/B2/C/D [%]	1/7/9/2 [5.3/36.8/47.4/10.5]	1/24/25/7 [1.8/42.1/43.9/12.3]	0/1/3/0 [0/25/75/0]	2/32/37/9 [2.5/40/46.3/11.3]

The Shapiro–Wilk test was used to confirm normal distribution. p≧ 0.05 was considered a normal distribution.

*ACVIM, classification based on Consensus Statements of the American College of Veterinary Internal Medicine (ACVIM) for myxomatous mitral valve disease in dogs: https://onlinelibrary.wiley.com/doi/10.1111/jvim.15488.

On arrival in Japan, four dogs showed severe symptoms related to heart failure. Three dogs were diagnosed with pulmonary edema (one dog classified as ACVIM stage C and two with ACVIM stage D), and one with left atrial rupture (classified as ACVIM stage B2). After receiving treatment, two dogs with pulmonary edema recovered, and two dogs died; thus, the overall survival rate for the inbound journey was 97.5%. The dog that died of pulmonary edema was a 7-year-old Cavalier King Charles Spaniel that flew from eastern USA (total flight hour, 12 h). The dog that died of left atrial rupture was a 9-year-old Maltese that flew from western USA (total flight hour, 11 h).

**Figure 1:**
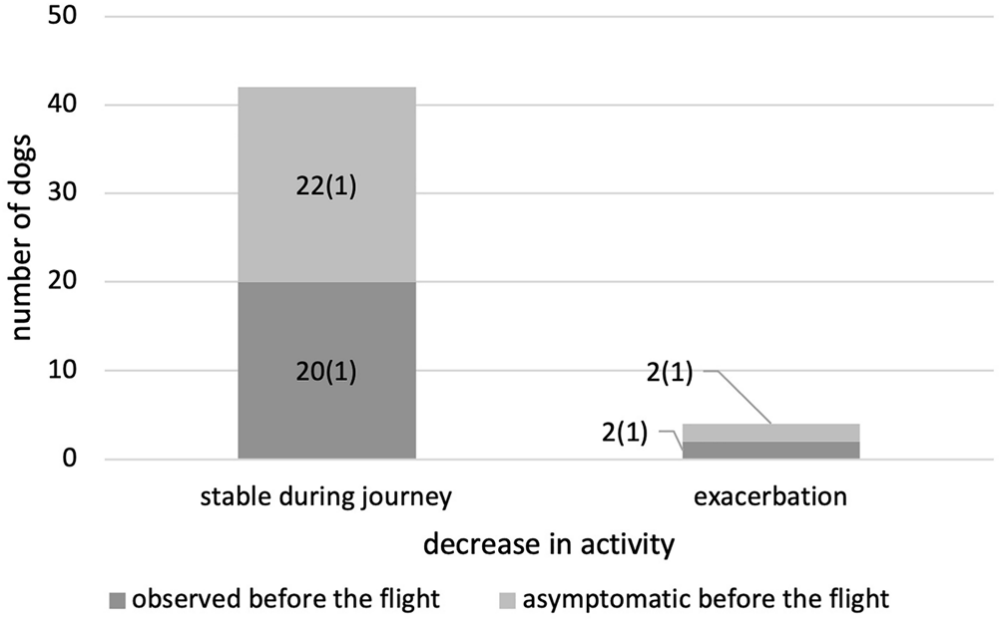
Decrease in activity status during the journey. The majority of dogs showed no changes in activity compared to the status before the flight. Numbers in parentheses represent the number of dogs diagnosed with cardiac events shortly after arrival.

**Figure 2:**
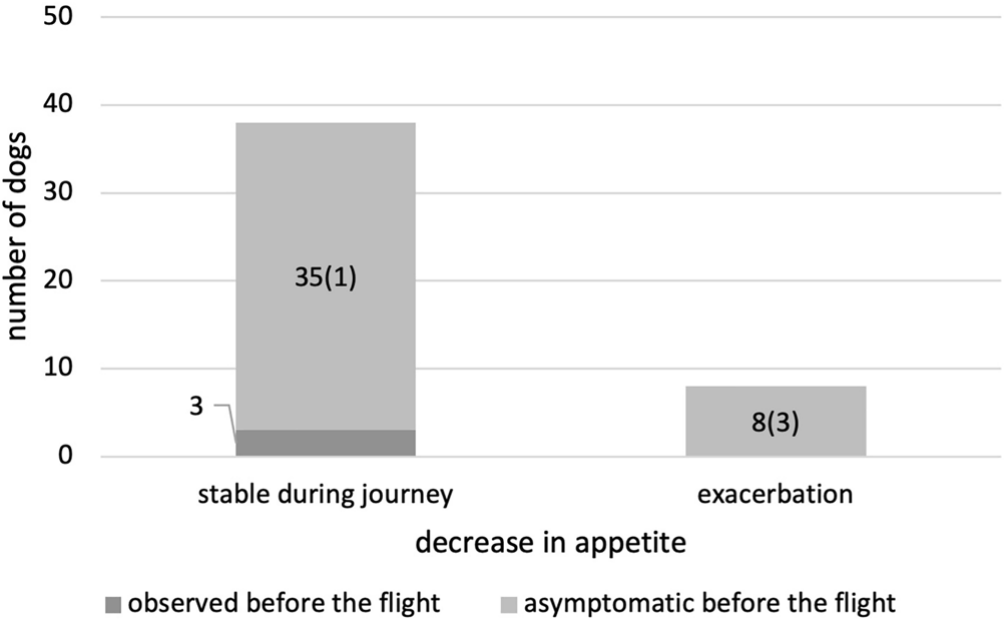
Decrease in appetite during the journey. The majority of dogs showed no changes in appetite compared to the status before the flight. Numbers in parentheses represent the number of dogs diagnosed with cardiac events shortly after arrival.

**Figure 3:**
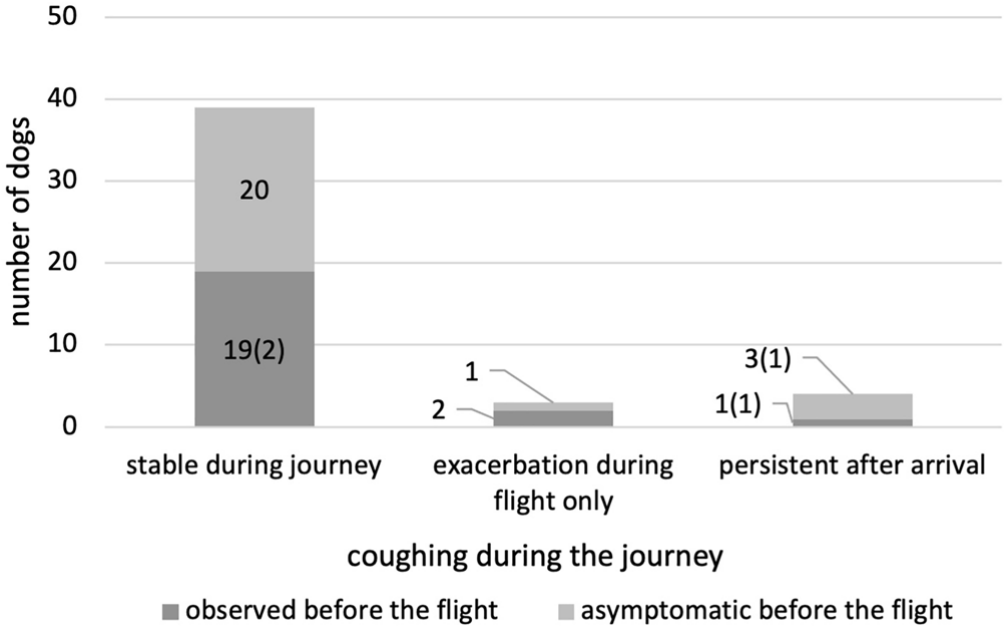
Coughing during the journey. The coughing status was stable during the journey in the majority of dogs, regardless of the symptoms prior to the flight. Exacerbation of coughing occurred in dogs in both the symptomatic and asymptomatic groups. Numbers in parentheses represent the number of dogs that showed cardiac events on arrival.

**Figure 4:**
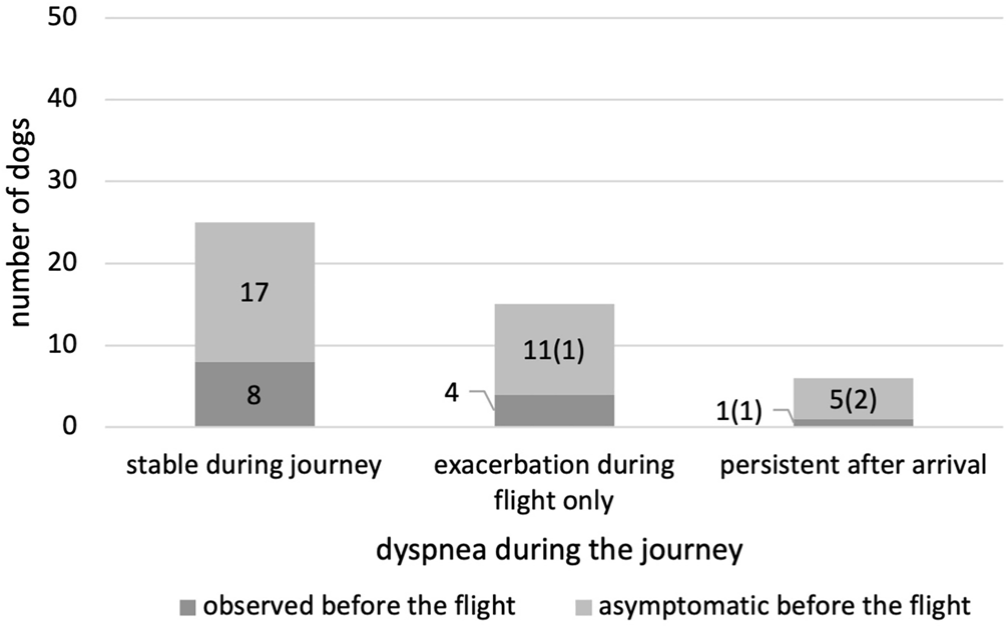
Dyspnea during the journey. More than half of the patients were able to travel in a stable respiratory state regardless of their abnormal respiratory state before the journey. In contrast, approximately half of the patients were suspected to have had a change in respiratory status due to travel. Of note, all dogs that had a cardiac event after the flight had an onset or exacerbation of dyspnea.

The conditions of the dogs during the journey were reported through an arbitrary questionnaire administered to owners. Of the 80 dogs included in this study, 46 completed the questionnaires. Of the 46 dogs, 47.8% (22) of the dogs showed decreased activity levels before air travel (Fig. 1). Two of these dogs reported a further decrease in activity during the flight. In addition, two dogs with normal activity levels before air travel were reported to have decreased activity during the journey. A reduction in appetite was reported in 6.5% (three) of the dogs before the flight, but these dogs were stable during the journey (Fig. 2). In contrast, of the 43 dogs that had a normal appetite before the air travel, eight dogs showed a decrease in appetite during the journey. Cough and dyspnea were monitored during the journey. Cough was observed in 47.8% (22) of the dogs before the flight (Fig. 3). Of these dogs, 86.4% showed stable status during the journey. Two dogs showed exacerbation of coughing only during the flight, and one dog that developed pulmonary edema showed persistent coughing after arrival. Of the 24 dogs that had no coughing symptoms before the flight, one dog was observed with the symptoms only during the flight, and three dogs showed persistent coughing after the flight (including one dog with pulmonary edema). Regarding dyspnea, 28.3% (13) of the dogs had symptoms prior to the flight (Fig. 4). Of these dogs, 61.5% had stable respiratory status during the journey. Four dogs showed exacerbation of dyspnea only during the journey, and one dog that developed pulmonary edema showed persistent symptoms after arrival. Among the 33 dogs with normal breathing status before the flight, 11 were reported to have dyspnea observed only during the flight (including one dog with pulmonary edema) and five dogs were reported to have persistent breathing problems after arrival (including two dogs with pulmonary edema). Digestive symptoms, such as vomiting and diarrhea, were not reported during the flight.

### Surgical survival rate and hospitalization period

Of the 80 dogs in this study, five did not undergo MVR. The reasons were death of two dogs after arrival, unfavorable preoperative screening results, or refusal of consent to surgery by the owner. Three of the 75 dogs that underwent MVR died postoperatively; thus, the surgical survival rate was 96%. In comparison, the surgical survival rate in 629 domestic dogs during the same study period at this facility was 94.3%. The average hospitalization periods were 7.3 days for the overseas dogs and 7.2 days for the domestic dogs.

### Pre- and post-air flight laboratory test results

To search for the risk factors for dogs with MMVD on air traveling, an attempt was made to compare the laboratory results before and after the flight. Blood tests, radiography, and echocardiographic data before the flight could only be collected from a small number of dogs. In addition, the most recent data varied from approximately 1 year to a few weeks prior to the flight. Considering the fact that MMVD is a progressive disease, data within 4 months prior to MVR were used, since this was the average period for domestic dogs with MMVD awaiting MVR after the date of surgery was appointed. The medical records of 10 dogs were used in this study. Values related to heart failure were assessed, including heart size from echocardiography and hematocrit, total protein, blood urea nitrogen, and creatinine levels from blood tests. Enlarged heart size based on the American College of Veterinary Internal Medicine (ACVIM) consensus guideline^7^ was recorded in all 10 dogs prior to the flight, but was confirmed in only seven dogs at the time of initial examination after arrival. Dehydration (diagnosed if two of the following three criteria were met: hematocrit > 55.0%, total protein > 7.2 g/dl, or blood urea nitrogen > 29.2 mg/dl in blood test results) was not observed in any of the dog prior to the flight and was confirmed in one dog after arrival. This dog was reported with a decrease in appetite during the flight from the owner survey. As for azotemia (defined as blood urea nitrogen > 29.2 mg/dl and creatinine > 1.4 mg/dl from blood tests), two dogs had persistent blood levels concerning azotemia, one resolved at the time of examination, and two developed after arrival. One of the dogs with an elevation in blood levels concerning azotemia was the dog with a decreased appetite. The second dog was reported with exacerbation of coughing and dyspnea during the flight. For the two dogs with persistent blood levels of azotemia, diarrhea or a decrease in activity was observed during the flight. No symptoms observed during the flight were of common occurrence with a specific laboratory result. No data prior to flight was available for the assessment of the two dogs that died after arrival.

## Discussion

In humans, a medical history of cardiac dysfunction, including valvular heart diseases, is considered one of the major factors that may lead to serious in-flight emergencies^4–6,8^. In-flight medical events reported in human studies include respiratory symptoms^4,8,9^, which were also observed in this study. An onset or exacerbation of dyspnea was observed in all four dogs that showed a cardiac event after arrival, although they were also reported in some dogs without critical conditions. In-flight symptoms observed during the flight varied among dogs, but some symptoms may have been overestimated or overlooked, as they were dependent on each owner’s subjective viewpoints. Despite this feature, the findings have provided a good insight for the owners to prepare when taking their dogs with MMVD overseas for treatment. The amount of data assessed may have increased if the questionnaire was mandatory.

The fact that cardiac enlargement showed recovery in some dogs compared to past medical data is presumably the result of interindividual variation in image assessment. Dehydration is considered to occur as humidity decreases at high altitudes^10^, which may be unfavorable for circulatory insufficiency. Only one dog showed dehydration in the blood test results after arrival. As most dogs maintain their appetite, dehydration may be prevented if access to water during flight is not restricted. The ideal goal is to set criteria based on clinical and laboratory findings for safe air travel for dogs with MMVD in need of MVR overseas. However, this was not achieved in the present study. Further risk assessments depending on cardiac failure status (i.e., ACVIM stage) or total flight hours will ensure less life-threatening air travel for these dogs.

The cause of critical conditions or death for MMVD dogs on arrival for the four dogs in this study was hypothesized to be related to the pathogenesis of MMVD because left atrial rupture and pulmonary edema can both be a result of left heart dysfunction leading to increased left atrial pressure. The exact trigger for the sudden change in the dog’s condition could not be verified by laboratory tests or owner’s statements. Additionally, the two dogs that died did not receive medication on their way to Japan. A conclusive discussion could not be made regarding medication compliance since information could not be obtained from all dogs. However, as medication plays an important role in controlling MMVD, failure in medication compliance may increase the risk of death for dogs with MMVD during traveling.

The surgical survival rate and hospitalization period for overseas dogs did not differ from those of domestic dogs. In addition, no emergency claims have been voluntarily reported to the authors for a return flight home for dogs that survived the MVR. This suggests that for dogs with MMVD, the risk of in-flight cardiac events decreases after MVR. These results imply that once a dog tolerates inbound flight, the effects are less likely to affect the surgical outcome. Therefore, taking MVR overseas may be included as an option to help dogs experiencing the disease, provided that the dogs are in a stable condition before the flight. The proportion of dogs showing fatal events seems rather low, and this report indicates that dogs with MMVD cannot be strongly prohibited from going onboard if MVR is included in the list of treatment options. At the same time, owners considering taking the chance to treat their dogs overseas should be aware of the in-flight symptoms and cardiac events behind them, as reported in some dogs in this study. It is strongly recommended that owners consult their local veterinarians in the short term before getting on board, adhering to the daily medication protocol while traveling, and have a medical check-up shortly after arrival in case their dogs experience any progressive symptoms. Further analysis is required to assure a safer air transportation for dogs with MMVD.

## Data Availability

All data generated or analyzed during this study are included in this published article.
